# High‐density mapping of de novo focal atrial tachycardias using a new software: Protected low‐voltage areas by zones of conduction delay

**DOI:** 10.1002/joa3.12380

**Published:** 2020-06-14

**Authors:** Konstantinos P. Letsas, Michael Efremidis, George Bazoukis, Stylianos Dragasis, Athanasia Megarisiotou, Panagiotis Mililis, Athanasios Saplaouras, Konstantinos Vlachos, Dimitrios Asvestas, Kosmas Valkanas, Gary Tse, Antonios Sideris

**Affiliations:** ^1^ Second Department of Cardiology Laboratory of Cardiac Electrophysiology “Evangelismos” General Hospital of Athens Athens Greece; ^2^ Xiamen Cardiovascular Hospital Xiamen University Xiamen P.R. China

**Keywords:** ablation, electroanatomical mapping, focal atrial tachycardia

## Abstract

**Background:**

The pathophysiological mechanism of focal atrial tachycardias (AT) remains obscure.

**Methods:**

Fifteen patients (6 males, age 45 ± 18) with focal AT underwent high‐density activation mapping using a new software called extended early‐meets‐late (EEML).

**Results:**

Irrespective of the arrhythmia mechanism, low bipolar voltage fractionated signals (0.14 ± 0.10 mV) were seen at the earliest activation site. The mean low‐voltage area (LVA) at the earliest activation site was 3.2 ± 1.0 cm^2^. EEML mapping revealed zones of conduction delay at the borders of LVAs.

**Conclusions:**

LVAs protected by zones of slow conduction appears to play an important role in the initiation and maintenance of focal ATs.

## Graphical abstract

Low‐voltage areas protected by zones of slow conduction appear to play an important role in the pathophysiology of focal ATs.

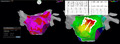

## 1 INTRODUCTION

Focal atrial tachycardias (AT) are characterized by a centrifugal atrial activation pattern irrespective of arrhythmia mechanism (microreentry, triggered activity, and enhanced automaticity).^1^ However, the pathophysiology of focal ATs is not fully elucidated. This study aimed to investigate the substrate of focal ATs during high‐density mapping using a novel software program.

## 2 PATIENTS AND METHODS

The study population consisted of 15 patients (10 males, age 45 ± 18 years) with an established diagnosis of focal atrial tachycardia based on electrophysiological criteria.^2^ None of these patients exhibited a history of atrial fibrillation or had undergone previous ablation procedure. The mechanism of AT was established based on certain criteria. The diagnosis of microreentrant AT was set up when activation mapping covered >70% of the tachycardia cycle length (TCL). Triggered activity was diagnosed when AT was inducible and terminated with programmed stimulation, and <50% of the TCL was mapped at the earliest activation site. Enhanced automaticity was diagnosed when AT was not inducible with programmed stimulation, and induction was achieved by isoproterenol infusion. Similar to triggered activity, <50% of the TCL was mapped at the earliest activation site. Adenosine testing was performed in selected cases.

The electrophysiological study was performed in fasting state without sedation. High‐density mapping of the atria was performed during AT to localize the site of origin using a three‐dimensional nonfluoroscopic mapping system (CARTO 3 Version 6.0, Biosense‐Webster). A new software of the mapping system [high‐definition (HD) coloring] identifies areas of potential conduction delay/block. The upper threshold of the early‐meets‐late tool is the same as in the previous software versions and is commonly used for mapping of macro‐ or microreentrant tachycardias (default upper threshold of 75%). A lower threshold has been added in CARTO 3 Version 6.0 system, called extended early‐meets‐late (EEML), which divides interpolated areas with significant activation time differences. EEML calculates the local activation difference between adjacent points, and if this difference is higher than the percentage selected (default lower threshold of 25%) of the cycle length mapped a white line will be drawn between those adjacent points indicating conduction delay or block. The system will highlight anywhere on the map where one area has at least a 25% difference from one color interpolation to another. Of note, the EEML is not dependent on the actual activation values themselves, but on the relative differences in activation time values used for color projection. A minimum of 1500 points was sampled throughout the right or left atrium using a multipolar catheter (PentaRay^®^ **catheter, number of electrodes 20, 2‐6‐2 mm interelectrode spacing, Biosense Webster). The earliest activation site was mapped with greater point density to delineate the extent and borders of the site of origin. The fill threshold for the electroanatomical mapping was set at 5. A contact force sensing catheter (SmartTouch, 8F, 3.5 mm tip electrode, 1‐6‐2 mm interelectrode spacing, Biosense Webster) was used for endocardial mapping and ablation (5‐15 g). Local abnormal bipolar electrograms were defined by those displaying low amplitude (<0.5 mV) and or long duration (>70 msec) with multicomponent potentials (>3 deflections). Confluent regions of bipolar low voltage were measured using the standard surface area measurement tool on the CARTO* *3* *software. Burst atrial stimulation with or without isoproterenol infusion up to 8 μg/min was used for arrhythmia induction in certain cases. The final confirmation of the AT site of origin was established during successful radiofrequency energy delivery (25‐35 W, 43°C).

Continuous data are presented as mean ± standard deviation. Categorical data are presented as frequency and percentage. The IBM SPSS program (Statistics for Windows, Version 22.0., IBM Corp) was used for the statistical analysis.

## 3 RESULTS

**FIGURE 1:**
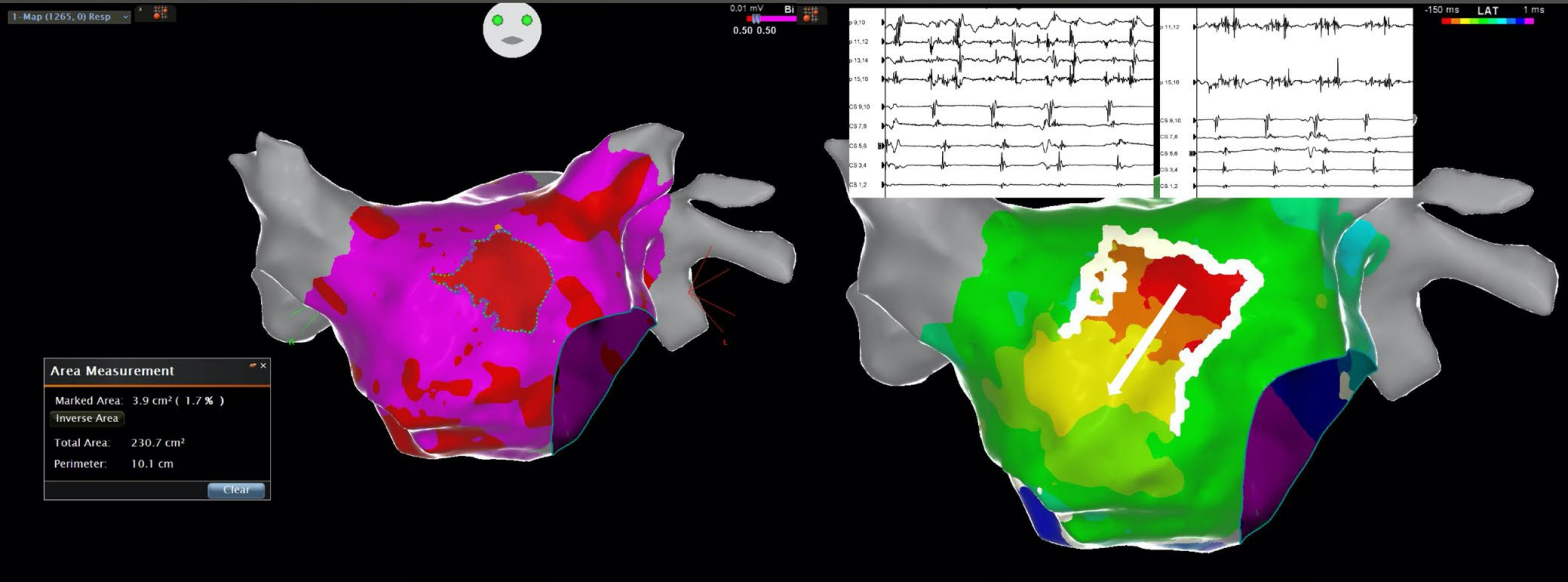
Left: Electroanatomical map showing an area (3.9 cm^2^) of low‐amplitude (<0.5 mV) bipolar signals at the earliest activation site. Right: Isochronal map demonstrating the earliest activation site, the zone of conduction block (white lines), and the preferential conduction canal (arrow) in a microreentrant focal AT arising from the left atrial septum. Low‐amplitude fractionated bipolar signals covering ≥70% of the tachycardia cycle length were recorded at the earliest activation site (p: signals recorded from the multipolar catheter at the earliest activation site; CS: signals recorded from coronary sinus catheter)

**FIGURE 2:**
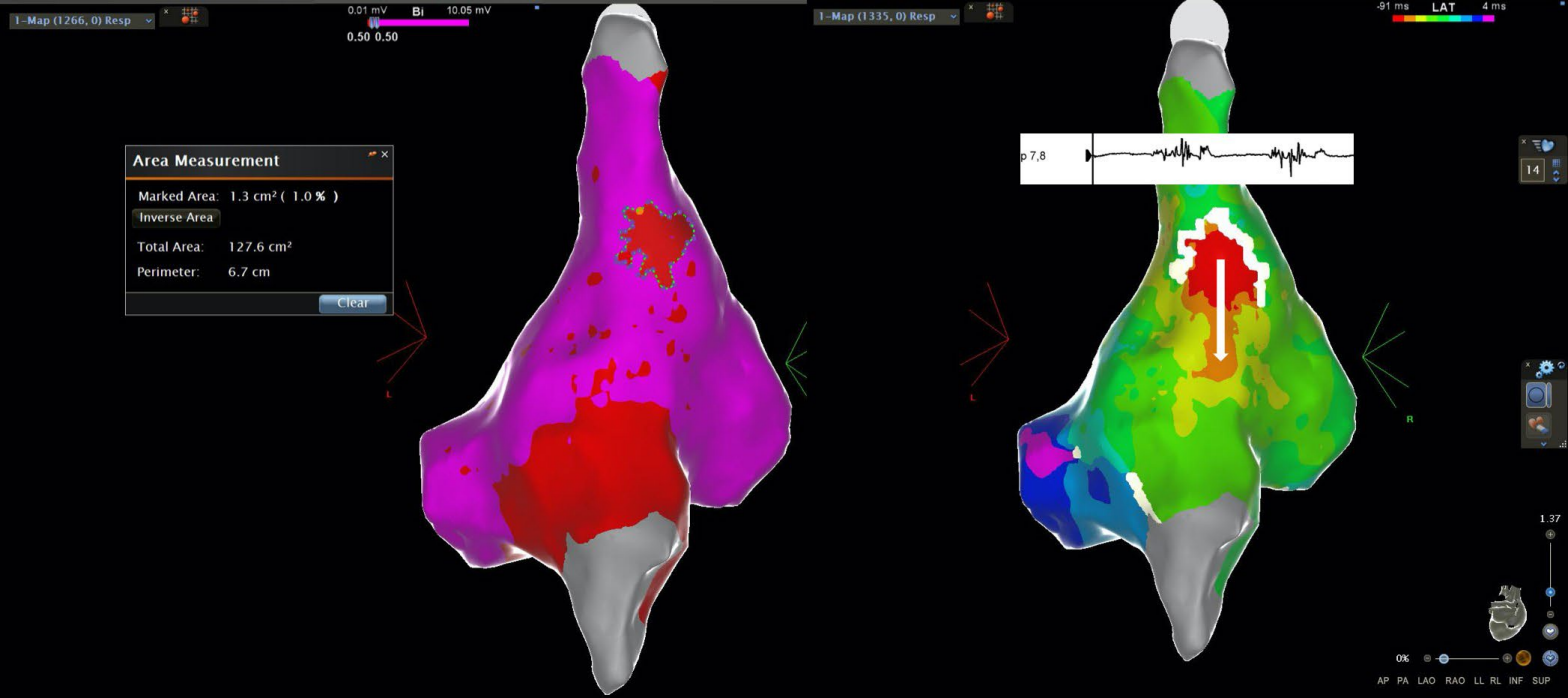
Left: Electroanatomical map showing a small area (1.3 cm^2^) of low‐amplitude (<0.5 mV) bipolar signals at the earliest activation site. Right: Isochronal map demonstrating the earliest activation site, the zone of conduction block (white lines), and the preferential conduction channel (arrow) in a case of triggered activity focal AT originating from the crista terminalis. Low‐voltage fractionated bipolar signals were recorded at the earliest activation site (p: signals recorded from the multipolar catheter at the earliest activation site)

The clinical characteristics, echocardiographic data, medications, and procedural data of the study cohort are depicted in Table 1. The underlying mechanism of the centrifugal ATs was triggered activity (n = 9), microreentry (n = 3), and enhanced automaticity (n = 3). The sites of origin included crista terminalis in 7, lateral right atrium in 2, ostium of the coronary sinus in 2, and left atrial septum in four patients. Adenosine testing when performed terminated triggered activity‐related ATs (n = 4), while no termination was seen in microreentrant‐related ATs (n = 2). Irrespective of the arrhythmia mechanism, very low‐voltage bipolar signals (0.14 ± 0.10 mV) with or without fractionation were mapped at the earliest activation site. The mean low‐voltage area was 3.2 ± 1.0 cm^2^. Apart from these low‐voltage areas at earliest activation site, the majority of patients (13/15) displayed normal electroanatomical bipolar voltage maps of the right or left atrium. All patients with microreentrant ATs demonstrated fractionated electrograms at the successful ablation site covering ≥70% of the TCL. EEML mapping revealed zones of conduction slowing/block (protected isthmus) around the earliest activation site in all subjects irrespective of arrhythmia mechanism. The zones of conduction slowing were located at the borders of the low‐voltage areas of the earliest activation site. A preferential conduction channel was evident in isochronal mapping in all cases (Figures 1 and 2). During a mean follow‐up of 7 ± 4 months, 14 of 15 patients were free of arrhythmias.

**TABLE 1:** Clinical characteristics, echocardiographic data, medications, and procedural data of the study cohort (n = 15)

Demographics	
Age (y)	45 ± 18
Gender (males)	10 (67%)
Medical history	
Hypertension	3 (20%)
Dyslipidemia	2 (13%)
Echocardiographic parameters	
LAD (mm)	34.0 ± 2.2
LVEF (%)	62.3 ± 4.9
Medications	
β‐blockers	12 (80%)
Calcium channel blockers	4 (27%)
Class IC antiarrhythmic drugs	7 (47%)
Procedural data	
Complications	0 (0%)
Procedure time (min)	107 ± 19
Arrhythmia mechanism	
Automaticity	3 (20%)
Triggered activity	9 (60%)
Microreentry	3 (20%)

Abbreviations: LAD, left atrial diameter; LVEF, left ventricular ejection fraction.

## 4 DISCUSSION

In this high‐density mapping study using a new software program, we demonstrated that focal ATs arise from low‐voltage areas that are protected by zones of conduction delay.

Earlier mapping studies with low sampling points have introduced the concept of “diseased areas” with preferential conduction channels in focal ATs. Marchlinski et al have initially reported focal ATs with preferential conduction using magnetic electroanatomical mapping.^3^ In a noncontact mapping study, focal depolarization from a low‐voltage area with preferential conduction and breakout site has been demonstrated in ATs.^4^

High‐density mapping with multielectrode catheters with smaller electrodes and closer interelectrode spacing allows better and detailed characterization of the substrate.^5^ Multielectrode catheters display higher sensitivity to near‐field signals allowing the identification of smaller low‐voltage areas and an increased amount of electrogram fractionation.^5^ EEML mapping is a new software modality allowing the direct visualization of zones of conduction delay/block, and providing a basis for better interpretation of isochronal and propagation maps. As stated, the EEML reflects relative differences in activation time values used for color projection, and is not dependent on the actual activation values themselves. Based on this fact, the EEML software is valid to detect conduction slowing irrespective of the arrhythmia mechanism. In our study, EEML mapping revealed zones of significant conduction delay around very low‐voltage areas (protected regions) displaying the earliest activation site. Preferential conduction channels along with a breakout site were identified by isochronal mapping in focal ATs. A recent study of 10 patients with left atrial tachycardia following extensive ablation including lines investigated the lower threshold settings of the HD Coloring algorithm for the detection of conduction delays. Settings of 20% or 15% have been shown to be more accurate than the default setting of 25% in displaying the actual amount of conduction block as assessed by ripple maps.^6^

The very low‐voltage areas of the foci possibly represent diseased fibrotic atrial tissue. Spach and Josephson reported reentrant circuits in humans that occur in very small areas (microreentry) because of nonuniform anisotropic conduction associated with microfibrosis.^7^ The zones of slow conduction at the borders of AT foci might be either functional or anatomical. Functional conduction delay is related to specific electrophysiological properties of the atrial myocardium involving dispersion of excitability or refractoriness as well as anisotropic conduction.^8^ Crista terminalis, a common location site of ATs, has been shown to display areas of prominent anisotropy with slow conduction properties.^9^ A preferential conduction that AT activation wavefronts pass through has been additionally demonstrated along or across the CT. Anatomic obstacles and/or anisotropic conduction is the most plausible explanation of these preferential conduction pathways.^9^

In conclusion, using EEML software, this high‐density mapping study demonstrated that focal ATs display a specific electroanatomical pattern involving a protected diseased atrial tissue by zones of slow conduction along with preferential conduction channels at the exit site. The thresholds of EEML software for the accurate detection of conduction blocks remain to be validated in further studies. An individual adjustment of the EEML thresholds may be applied for a more accurate visualization of the zones of conduction block.

## CONFLICT OF INTEREST

None to declare.
